# Syndecan-2 Is a Novel Target of Insulin-Like Growth Factor Binding Protein-3 and Is Over-Expressed in Fibrosis

**DOI:** 10.1371/journal.pone.0043049

**Published:** 2012-08-10

**Authors:** Ximena D. Ruiz, Logan R. Mlakar, Yukie Yamaguchi, Yunyun Su, Adriana T. Larregina, Joseph M. Pilewski, Carol A. Feghali-Bostwick

**Affiliations:** 1 Division of Pulmonary, Allergy, and Critical Care Medicine, Department of Medicine, University of Pittsburgh School of Medicine, Pittsburgh, Pennsylvania, United States of America; 2 Department of Dermatology, University of Pittsburgh School of Medicine, Pittsburgh, Pennsylvania, United States of America; 3 Department of Pathology, University of Pittsburgh School of Medicine, Pittsburgh, Pennsylvania, United States of America; Cedars-Sinai Medical Center, United States of America

## Abstract

Extracellular matrix deposition and tissue scarring characterize the process of fibrosis. Transforming growth factor beta (TGFβ) and Insulin-like growth factor binding protein-3 (IGFBP-3) have been implicated in the pathogenesis of fibrosis in various tissues by inducing mesenchymal cell proliferation and extracellular matrix deposition. We identified Syndecan-2 (SDC2) as a gene induced by TGFβ in an IGFBP-3-dependent manner. TGFβ induction of SDC2 mRNA and protein required IGFBP-3. IGFBP-3 independently induced production of SDC2 in primary fibroblasts. Using an *ex-vivo* model of human skin in organ culture expressing IGFBP-3, we demonstrate that IGFBP-3 induces SDC2 *ex vivo* in human tissue. We also identified Mitogen-activated protein kinase-interacting kinase (Mknk2) as a gene induced by IGFBP-3. IGFBP-3 triggered Mknk2 phosphorylation resulting in its activation. Mknk2 independently induced SDC2 in human skin. Since IGFBP-3 is over-expressed in fibrotic tissues, we examined SDC2 levels in skin and lung tissues of patients with systemic sclerosis (SSc) and lung tissues of patients with idiopathic pulmonary fibrosis (IPF). SDC2 levels were increased in fibrotic dermal and lung tissues of patients with SSc and in lung tissues of patients with IPF. This is the first report describing elevated levels of SDC2 in fibrosis. Increased SDC2 expression is due, at least in part, to the activity of two pro-fibrotic factors, TGFβ and IGFBP-3.

## Introduction

The process of fibrosis is characterized by activation and proliferation of fibroblasts, and excessive deposition of extracellular matrix (ECM) that produces abnormal scarring of tissues leading to organ failure. The balance between pro-fibrotic and anti-fibrotic factors plays an important role in the development of fibrosis. Transforming growth factor beta (TGFβ) is one of the most studied pro-fibrotic factors. It has been implicated in the pathogenesis of liver, kidney, lung and skin fibrosis [1]. TGFβ induces mesenchymal cell proliferation, increased ECM production and a fibrotic response in various tissues [2].

We and others have shown that TGFβ induces Insulin like growth factor binding protein-3 (IGFBP-3) mRNA and protein expression [3], [4], [5], [6], [7], [8]. In previous studies, we demonstrated a time-dependent increase in IGFBP-3 secretion in response to TGFβ stimulation of primary lung fibroblasts [8]. IGFBPs are carrier proteins that can exert their function through Insulin-like growth factors (IGF). IGFBP-3 also has IGF-independent effects that involve interaction with TGFβ receptors and direct translocation into the nucleus [9], [10]. IGFBP-3 levels are increased in the bronchoalveolar lavage (BAL), lung tissue, and primary fibroblasts of patients with idiopathic pulmonary fibrosis (IPF) [8], [11]. We have shown that IGFBP-3 contributes to ECM deposition in primary fibroblasts and increases dermal and collagen bundle thickness in a human *ex vivo* skin explant model [8], [12], [13].

Using microarray analysis (unpublished data), we identified Syndecan-2 (SDC2) as a TGFβ-induced gene requiring IGFBP-3 expression. TGFβ has been shown to induce heparan sulfate proteoglycan (HSPG) production independently of its effects on proliferation. TGFβ also up-regulates proteoglycan production in the bleomycin model of lung fibrosis [14], and induces SDC2 expression in human periodontal ligament cells, osteoblasts and rat liver fibroblasts [15], [16], [17]. Using the same microarray analysis, we also identified Mitogen-activated protein kinase-interacting kinase (Mknk2) as a gene induced by IGFBP-3. Two isoforms of Mknk2 have been identified, Mknk2a and Mknk2b [18]. We show that IGFBP-3 specifically induced Mknk2a levels and phosphorylation. Mknk2 acted downstream of IGFBP-3 to induce SDC2 production *ex vivo*.

SDC2 is a HSPG expressed in endothelial, mesenchymal and carcinoma cells. HSPGs sequester proteins within secretory vesicles, link proteins together within the ECM, and bind proteins to the cell surface. The Syndecans are a family of four transmembrane proteoglycans divided in two subfamilies (Syndecan 1 and 3 and Syndecan 2 and 4, respectively) based on their sequence homology [19]. A cytoplasmic domain enables the Syndecans to associate with cytoskeletal proteins and signaling molecules [20]. The SDC2 ectodomain promotes focal adhesion and stress fiber formation in fibroblasts in a distinct pattern from fibronectin and independent of heparan sulfate requirement [21]. SDC2 also controls laminin and fibronectin matrix assembly into a fibrillar matrix [22], [23]. SDC2 regulates TGFβ induction of matrix deposition and increases total and surface levels of TGFβ receptors type I and II [24]. SDC2 expression is increased in the tubulo-interstitium of kidneys from type II diabetic patients, and its inducible role in fibrosis has been demonstrated in Syndecan 4 deficient mice, where SDC2 is up-regulated in parallel with TGFβ during unilateral nephrectomy-induced glomerulosclerosis [24], [25]. SDC2 also increases Integrin-alpha2 expression levels and enhances collagen adhesion, cell migration and invasion in normal rat intestinal epithelial cells [26]. The importance of SDC2 in matrix remodeling was emphasized by silencing SDC2, which resulted in disruption of actin cytoskeleton formation and fibronectin deposition [27].

**Figure 1:**
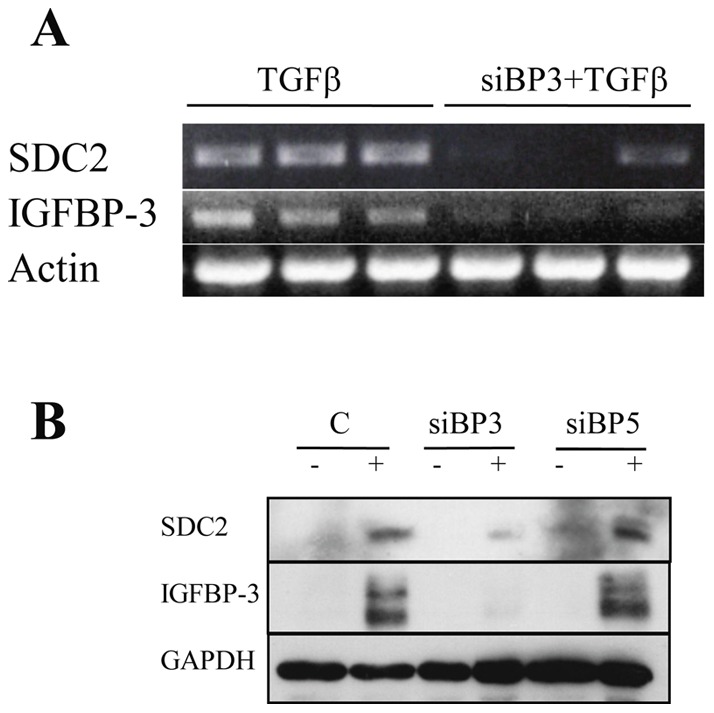
A) TGFβ induction of SDC2 gene expression is IGFBP-3-dependent. Normal fibroblasts were transfected with siRNA targeting IGFBP-3 (siBP3), then stimulated with TGFβ (10 ng/ml) for 48 hours. RT-PCR was used for the detection of SDC2 in 100 ng equivalent of template. β-actin was used as control. Experiments were done in triplicate. B) TGFβ induction of SDC2 protein is IGFBP-3-dependent. Primary human lung fibroblasts were transfected with siRNA targeting IGFBP-3 or IGFBP-5 as a related protein control. Fibroblasts were stimulated with TGFβ (10 ng/ml). Cellular lysates were analyzed by immunoblotting for SDC2 protein after 72 hrs. Efficiency of silencing was assessed by detecting IGFBP-3. GAPDH was used as internal control.

In summary, we identified SDC2 as a novel target of IGFBP-3 and TGFβ. Both IGFBP-3 and TGFβ induce SDC2 expression, and TGFβ induction of SDC2 is mediated by IGFBP-3. SDC2 is also over-expressed in fibrotic dermal and lung tissues. Our findings identify a novel pathway involving TGFβ, IGFBP-3, Mknk2 and SDC2 in organ fibrosis.

## Results

### TGFβ1 induction of SDC2 gene expression is mediated by IGFBP-3

SDC2 was identified by microarray analysis as a gene induced by TGFβ in an IGFBP-3-dependent manner. Briefly, IGFBP-3 was silenced in primary lung fibroblasts using sequence-specific siRNA, then cells were stimulated with TGFβ to assess which genes require IGFBP-3 for induction by TGFβ (data not shown). To confirm the microarray findings we repeated the experiment and examined SDC2 mRNA expression. When IGFBP-3 was silenced using siRNA, TGFβ induction of SDC2 gene expression was abolished, suggesting that TGFβ requires IGFBP-3 for the induction of SDC2 (Figure 1A). Silencing IGFBP-3 did not alter mRNA levels of SDC1, SDC3 or SDC4 (Figure S1).

We have previously shown that IGFBP-3 secretion is increased in fibrosis [8]. Since both TGFβ and IGFBP-3 are implicated in the development of fibrosis, and both of these factors induce expression of SDC2 mRNA (Figure 1A), we assessed SDC2 protein levels in primary lung fibroblasts stimulated with TGFβ and in which IGFBP-3 expression and induction by TGFβ were silenced. TGFβ induction of SDC2 protein levels was also dependent on IGFBP-3 as silencing of IGFBP-3 abolished TGFβ induction of SDC2 (Figure 1B).

### IGFBP-3 can directly induce SDC2 expression

**Figure 2:**
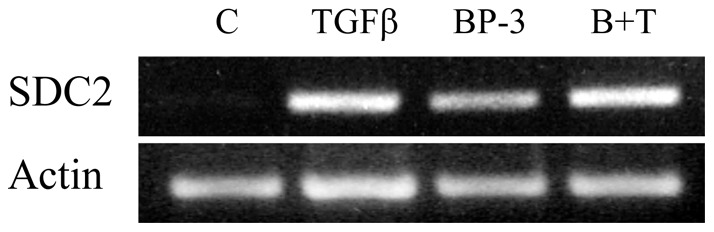
TGFβ and IGFBP-3 induce SDC2 gene expression. Normal lung fibroblasts were stimulated with recombinant TGFβ (10 ng/ml), IGFBP-3 (BP-3; 250 ng/ml) or both (B+T) for 48 hours. SDC2 mRNA was detected by RT-PCR using 100 ng template. β-actin was used as control.

To determine if IGFBP-3 can stimulate SDC2 expression independently of TGFβ, we stimulated primary lung fibroblasts with recombinant TGFβ, IGFBP-3, or both growth factors and examined SDC2 mRNA levels. *In vitro*, TGFβ and IGFBP-3 induced SDC2 gene expression independently, but their combined use did not result in an additive or synergistic effect (Figure 2).

### IGFBP-3 induces SDC2 gene and protein expression in a time-dependent manner

**Figure 3:**
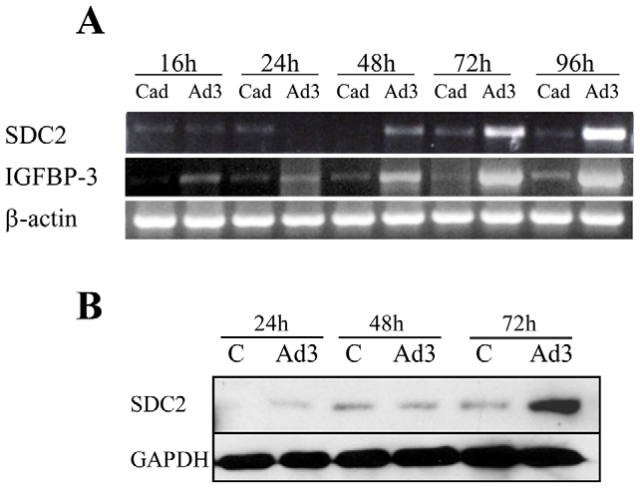
IGFBP-3 induces SDC2 expression in a time-dependent manner. Primary fibroblasts were infected with Ad-IGFBP3 (Ad3) or control Ad (Cad) at an MOI of 50 for 24 h, 48 h, 72 h and 96 h respectively. **A**) SDC2 and IGFBP-3 gene expression was examined by RT-PCR. β-actin was used as control. **B**) SDC2 protein levels were detected by immunoblotting. GAPDH was used as control.

Having shown that recombinant IGFBP-3 can independently induce SDC2 expression, we confirmed our findings and extended them using adenoviral expression of IGFBP-3 in primary human fibroblasts. Adenoviral expression of IGFBP-3 increased SDC2 mRNA levels in a time-dependent manner (Figure 3A). In contrast to induction of SDC2, expression of IGFBP-3 did not increase expression of SDC1, SDC3, or SDC4 (Figure S2), and in fact a reduction in SDC4 mRNA was noted at 24 and 48 hrs. Adenoviral expression of IGFBP-3 also resulted in increased production of SDC2 protein in culture media conditioned by primary human fibroblasts (Figure 3B).

### SDC2 production is induced in skin engineered to express IGFBP-3

**Figure 4:**
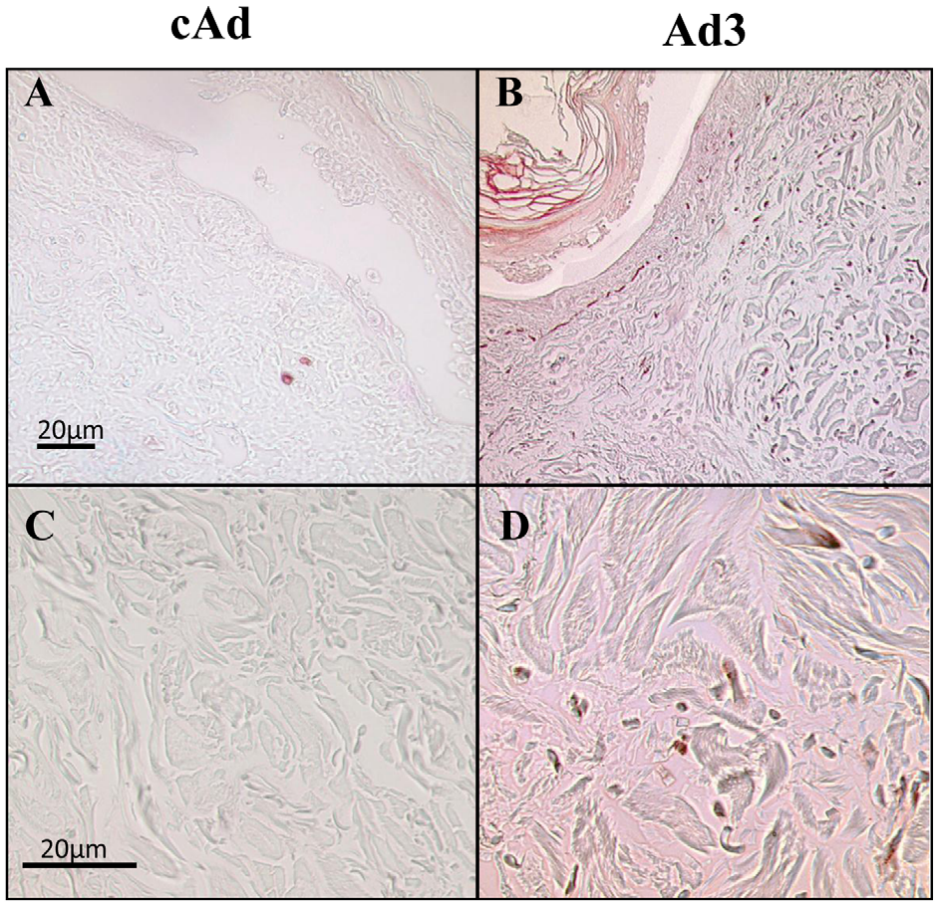
SDC2 is over-expressed in IGFBP-3 expressing human skin. Human skin explants were infected with either control adenovirus (cAd; **A** and **C**) or adenovirus encoding IGFBP-3 (Ad-3; **B** and **D**) and maintained in culture for 7 d and sections of paraffin embedded tissue were analyzed for the expression of SDC2 by immunohistochemistry. **B** and **D** are histological images showing the expression of SCD2 in dermal fibroblasts following infection with Ad-IGFBP-3. A and B, magnification = 100x. C and D, magnification = 400x.

We have previously demonstrated that IGFBP-3 can induce a fibrotic phenotype *in vitro*, *in vivo*, and *ex vivo* [8], [12], [13]. To assess the effects of IGFBP-3 in human tissues, we detected SDC2 proteins in human skin maintained in organ culture and engineered to express human IGFBP-3 as previously described [12]. Using immunohistochemistry, we detected an increase in SDC2 expression in the upper dermis of skin injected with IGFBP-3 expressing adenovirus (Figure 4).

### SDC2 is over-expressed in fibrotic tissues

**Figure 5:**
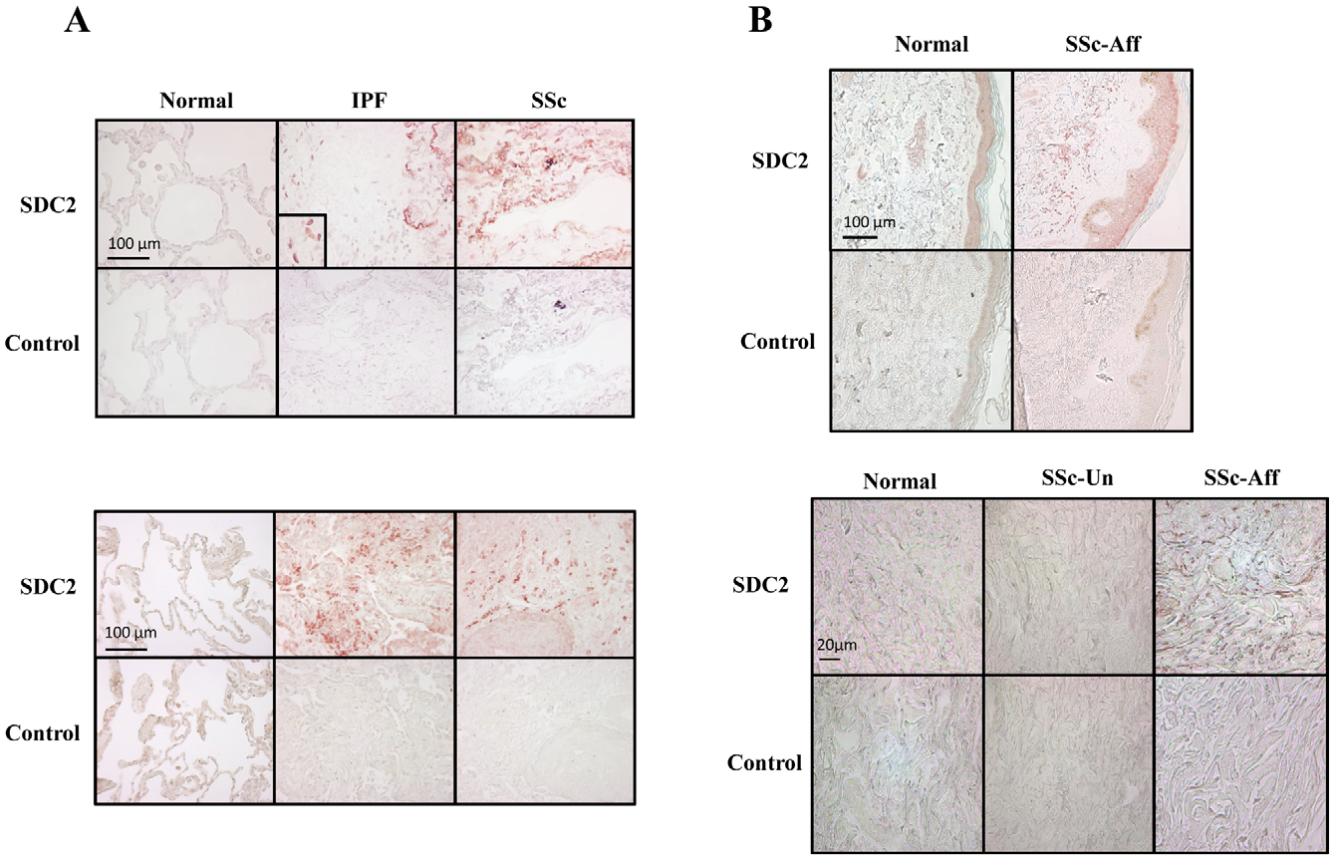
A) SDC2 is highly expressed in fibrotic lung. Immunohistochemistry was used to detect SDC2 in lung tissues normal donors, patients with IPF, and patients with SSc-associated pulmonary fibrosis. Images are representative of data obtained with lung tissues from 6 normal donors, 9 patients with IPF, and 6 patients with SSc. Rabbit IgG was used as an antibody control. Magnification = 400x. B) SDC2 is over-expressed in SSc affected skin. Immunohistochemistry was used to detect SDC2 in normal donor skin, clinically unaffected and affected skin from a patient with SSc. Images are representative of data from skin of 4 patients with SSc and two controls. Magnification = 200x (left panel), 400x (right panel).

Since IGFBP-3 induces SDC2 expression and IGFBP-3 expression and deposition is increased in IPF [8], we used immunohistochemistry to detect SDC2 protein *in vivo* in IPF lung tissues and those from patients with SSc-associated pulmonary fibrosis. Compared to normal lung, SDC2 protein was increased in fibrotic lung tissues of patients with IPF and SSc-associated pulmonary fibrosis (Figure 5A). To determine if increased SDC2 in lung tissues is organ-specific, we examined skin tissues from patients with SSc. SDC2 was also increased in the clinically affected skin of patients with SSc compared with clinically unaffected skin from the same patients and normal donor skin (Figure 5B).

### Silencing SDC2 does not prevent TGFβ induction of fibrotic genes

Since SDC2 is increased in fibrotic organs, we examined the effect of silencing SDC2 in primary human fibroblasts on the levels of TGFβ-inducible genes. SDC2 silencing did not significantly alter TGFβ induction of collagen, fibronectin, αSMA, or CTGF in primary fibroblasts from two donors and in MRC-5 cells (Figures S3A–S3C). This may be due, in part, to the long half-life of HSPGs compared to the relatively short duration of *in vitro* silencing experiments.

### IGFBP-3 activates Mknk2

**Figure 6:**
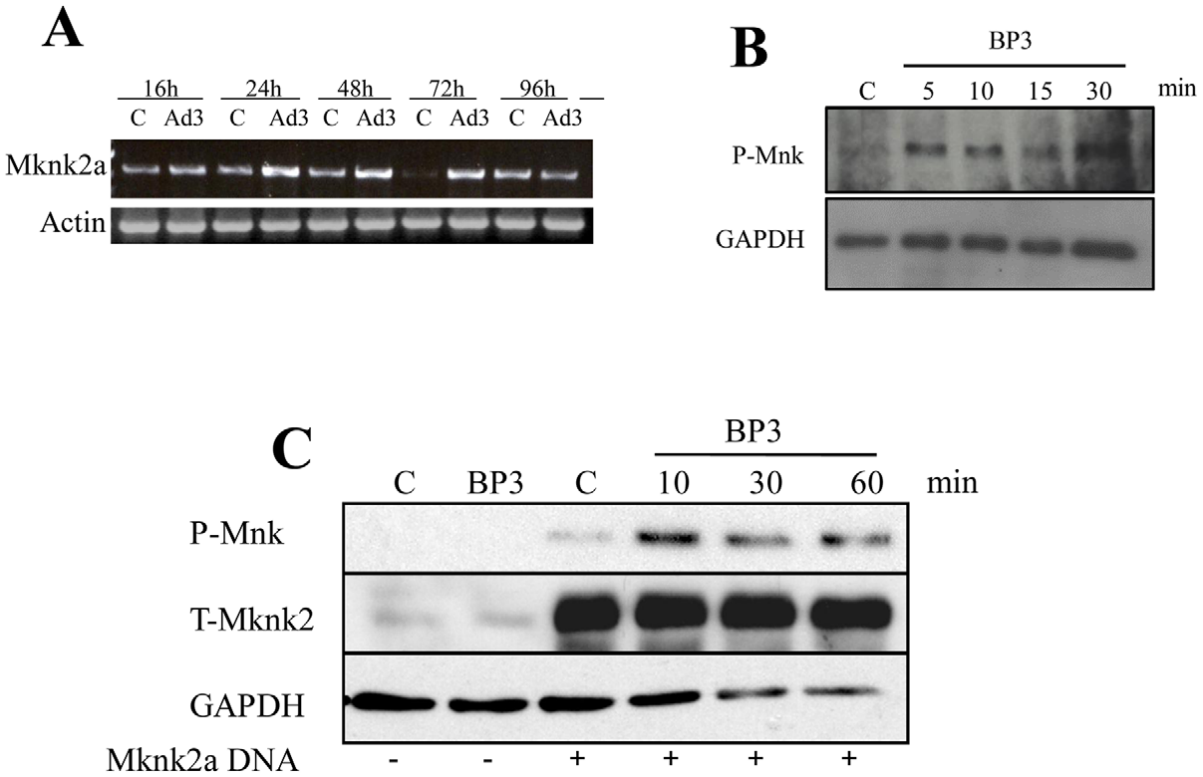
IGFBP-3 induces Mknk2a expression and activation. Primary normal skin fibroblasts were infected with Ad-IGFBP3 (Ad3) or control Ad (C) at an MOI of 50 for 24 h, 48 h and 72 h or 48 h, 72 h and 96 h. **A**) Mknk2a gene expression was examined by RT-PCR. **B**) **IGFBP-3 activates Mnk.** Primary human lung fibroblasts were treated with 250 ng/ml recombinant human IGFBP-3 for 5–30 minutes. Phosphorylation of Mnk was assessed by immunoblotting. **C**) **IGFBP-3 activates Mknk2a.** MRC-5 cells were transfected with p-Adlox expressing human Mknk2a. Cells were stimulated with rhIGFBP-3 (250 ng/ml). Lysates were examined for Mknk2 activation at 10, 30 and 60 minutes. Total Mknk2 and GAPDH were used as loading controls.

IGFBP-3 activates p44/42 MAPK signaling cascade [28]. One of the genes identified by microarray analysis to be induced by IGFBP-3 was Mknk2 (data not shown), a downstream target of p44/42 MAPK [18]. To confirm the microarray data, we examined mRNA and protein levels of Mknk2 following IGFBP-3 expression. IGFBP-3 caused a dose-dependent increase in Mknk2a expression (Figure 6A) and an increase in Mnk phosphorylation (Figure 6B). To confirm the phosphorylation of Mknk2a, we generated a construct expressing human Mknk2a and confirmed the activation of Mknk2a by IGFBP-3 in primary human fibroblasts. Mknk2 was phosphorylated in fibroblasts expressing human Mknk2a and stimulated with recombinant IGFBP-3 (Figure 6C). Thus, IGFBP-3 increases levels and activation of Mknk2a in human fibroblasts.

### Mknk2a induces SDC2 production

**Figure 7:**
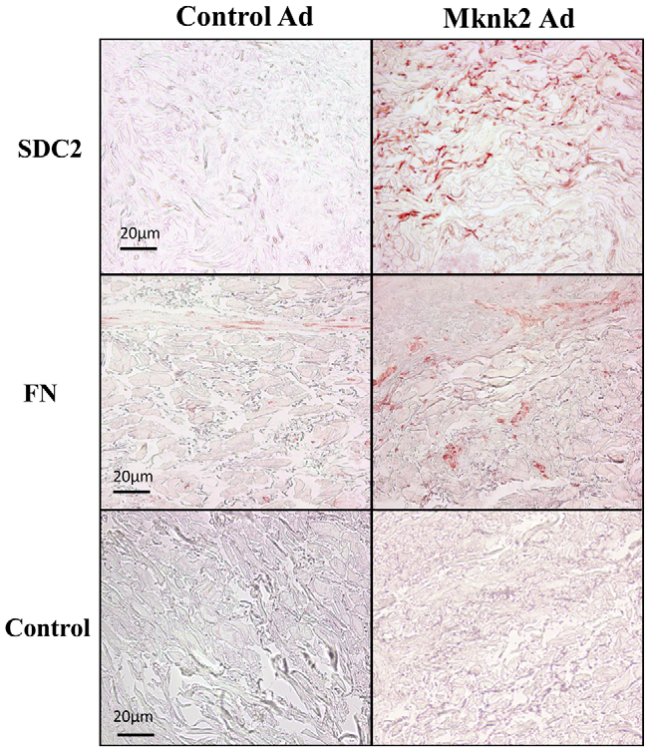
SDC2 levels increase in human skin engineered to express Mknk2a. Human skin was injected with cAd or Ad-Mknk2a and maintained in organ culture for one week. SDC2 and fibronectin were detected by immunohistochemistry. A control antibody was used in the lower panels. Magnification = 200x.

To assess the effect of Mknk2a on SDC2 production in human tissues, SDC2 was detected in human skin engineered to express Mknk2a. Mknk2a induced expression of SDC2 as detected by immunohistochemistry (Figure 7). Induction of SDC2 was specific as a parallel increase in fibronectin levels was not observed (Figure 7). In contrast, we have previously shown that IGFBP-3 induces expression of fibronectin in human skin [12]. This suggests that Mknk2a likely mediates IGFBP-3 induction of SDC2 but not fibronectin.

## Discussion

IGFBP-3 and TGFβ have been implicated in the development of fibrosis in SSc and IPF, as well as other fibrosing conditions. We and others have shown that TGFβ increases IGFBP-3 secretion *in vitro* [3], [4], [5], [6], [7], [8]. We now demonstrate that IGFBP-3 independently induces SDC2 and regulates TGFβ induction of SDC2 in primary human fibroblasts. Thus IGFBP-3 serves as a downstream modulator of TGFβ action. Our data also show that SDC2 levels are increased *in vivo* in fibrotic lung and skin.

It is well documented that IGFBP-3 activates the MAPK signaling cascade. A component of the cascade, p44/42 MAPK is reported to phosphorylate and thus activate the Mnk family of kinases that includes Mnk1 and Mknk2a and 2b [18]. We show that IGFBP-3 induces Mknk2a expression and results in the activation of this kinase. Mknk2a in turn induces SDC2 production in human skin engineered to express the kinase. Mknk2a exerts some of its effects in mammalian cells via phosphorylation of eukaryotic initiation factor 4E, thus modulating protein translation [29], [30], [31]. Although the exact mechanism by which Mknk2a modulates SDC2 levels remains to be determined, our findings suggest that IGFBP-3 triggers MAPK activation of Mknk2a, which then mediates its stimulatory effects on SDC2 production.

The syndecans, including SDC2, mediate several cellular processes including cell signaling and cytoskeletal organization. SDC2 promotes cell proliferation and migration. SDC2 also mediates cell-matrix interactions. SDC2 is over-expressed in fibrotic glomerulosclerosed kidneys of Syndecan-4 null mice and fibroblasts from the tubulointerstitium of kidneys from type II diabetic patients [23], [24]. We identified that SDC2 is over-expressed in fibrotic skin of patients with SSc as well as fibrotic lung of SSc and IPF patients. Our findings and those of others suggest that SDC2 is increased in fibrosis irrespective of the organ or fibrotic trigger involved.

SDC2 is induced by TGFβ, but *in vitro* silencing SDC2 does not abrogate TGFβ induction of fibrotic genes such as collagen, fibronectin and CTGF. This is not surprising since the kinetics of TGFβ induction of SDC2 parallel those of other fibrotic genes and since HSPG's are known to have a prolonged half-life. However, we can envision other roles for SDC2 in fibrosis, based on its function in tumorigenesis and metastasis. SDC2 serves as a docking receptor for matrix metalloproteinase (MMP)-7 in cancer cells [32], an enzyme implicated in the pathogenesis of fibrosis [33], [34]. SDC2 also suppresses MMP-2 activation, and subsequently metastasis, of lung carcinoma cells [35]. In fibrosis, a reduction in MMP-2 activity would result in decreased matrix degradation and increased accumulation of extracellular matrix proteins.

An additional potential role for SDC2 in fibrosis emerges from its role in extracellular matrix assembly [22]. We show that SDC2 expression is induced by IGFBP-3 and TGFβ. Since both TGFβ and IGFBP-3 induce fibronectin production, and SDC2 is required for fibronectin matrix assembly, SDC2 may contribute to fibrosis by enhancing ECM assembly and deposition. Modulation of TGFβ action is another mechanism by which SDC2 could potentially act, since it was reported to potentiate TGFβ mediated matrix deposition by increasing the amounts of types I and II TGF receptors [24]. SDC2 also interacts with growth factors such as members of the TGFβ superfamily and sequesters them to present them to their receptor(s) and even functions as a co-receptor for various factors [36]. Furthermore, SDC2 has been shown to regulate cell migration by enhancing adhesion and proliferation of cancer cells and fibroblasts [37], [38]. Enhanced migration and proliferation of fibroblasts is another mechanism implicated in the pathogenesis of organ fibrosis.

Although SDC-1 and -4 have been implicated in fibrosis [39], [40], [41], [42], [43] and have been suggested as targets for the treatment of lung diseases [44], we did not detect an increase of SDC-1, 3, or 4 in primary human fibroblasts in response to TGFβ or IGFBP-3. We demonstrate increased SDC2 levels in human fibrotic diseases as well as regulation of SDC2 by IGFBP-3. Further experimentation is warranted to determine whether regulating SDC2 can interfere with matrix assembly in fibrosis.

## Materials and Methods

### Materials, Antibodies and Reagents

Dulbecco's modified Eagle's medium (DMEM) was purchased from Cellgro-Mediatech, Inc (Herndon, VA), fetal bovine serum (FBS) from Sigma-Aldrich (St. Louis, MO), penicillin, streptomycin, and anti-mycotic agents from Invitrogen Life Technologies (Carlsbad, CA). The Aminoethyl Carbazole Substrate kit was from Zymed (San Francisco, CA). The ABC kit was from Vector labs, Inc. (Burlingame, CA). TRIzol, oligo (dT)12_15 primer, Superscript II reverse transcriptase, Protein A, and Protein G agarose were from Invitrogen Life Technologies. Antibodies were purchased as follows: Anti-human GAPDH, anti-fibronectin, and anti-human IGFBP-3 antibodies were purchased from Santa Cruz, Inc. (Santa Cruz Biotechnology, Inc., CA). Anti-human Syndecan-2 was purchased from Zymed. Anti-human Mknk2 was purchased from Sigma (St Louis, MO), Anti-phospho-mnk1 was purchased from Cell Signaling (Beverly, MA). Anti-CTGF and α-SMA antibodies were purchased from Abcam (Cambridge, MA), and Anti-collagen 1A1 was from Abnova (Taipei, Taiwan). Species-specific horseradish peroxidase-conjugated secondary antibodies were purchased from Santa Cruz, Inc. and GE Healthcare (UK). Recombinant human (rh) IGFBP-3 was purchased from Gropep Ltd (Adelaide, Australia) and rh-TGFB1 from R&D Systems (Minneapolis, MN).

### Cell Culture and Treatment

All tissues were obtained under a protocol approved by the Institutional Review Board of the University of Pittsburgh and with the written consent of donors or donor families (for normal lung donors). Primary human fibroblasts were obtained from healthy control donors and patients with systemic sclerosis and idiopathic pulmonary fibrosis. Lung fibroblasts were obtained from lung explants of patients undergoing lung transplantation at the University of Pittsburgh Medical Center. MRC-5 cells were obtained from American Type Culture Collection (Manassas, VA). Cells were grown in Dulbecco's modified Eagle's medium (DMEM) supplemented with 10% fetal bovine serum (FBS), 100 U/ml penicillin, 10 mg/ml streptomycin and 2.5 mg/ml amphotericin B, and maintained at 37°C in 5% CO2 humidified incubator. Primary fibroblasts were used in passages 2 to 7. For RNA silencing, primary fibroblasts in early passage were plated at 1.5×10^5^ in 6-well culture plates. Fibroblasts were transfected with 100 pmol sequence-specific or scrambled control siRNA (Life Technologies (Grand Island, NY) using Lipofectamine 2000 (Invitrogen Life Technologies, Inc.). Cells were harvested for RNA or protein extraction after 48 hrs. For treatment with recombinant proteins, cells were serum starved 24 h in serum free media, then rh-IGFBP-3 (250 ng/ml) or rhTGFβ (10 ng/ml) were added for 24–72 hours. For adenoviral gene expression, infections were carried out in PBS with Control (cAd), IGFBP3 (Ad-IGFBP3) or Mknk2a expressing adenovirus at a multiplicity of infection (MOI) of 50. Briefly, fibroblasts were incubated with adenovirus for 1 h at room temperature and then in growth medium for 16 to 96 hours. Culture supernatants, extracellular matrix and cellular lysates were harvested at the indicated times for each experiment.

### RNA Extraction and Reverse Transcription-PCR (RT-PCR)

Total RNA from cultured cells was extracted using Trizol reagent (Life Technologies, Inc.) according to the manufacturer instructions. Two µg of total RNA were used as template for reverse transcription using random primers. The cDNA obtained was amplified using the following primers: β-actin forward primer *5′-atgtttgagaccttcaacac-3′* and reverse primer *5*′*-cacgtcacacttcatgatgg-3*′; GAPDH forward primer 5′- cgaccactttgtcaagctca -3′ and reverse primer 5′- aggggtctacatggcaactg -3′; SDC1 forward primer 5′- gggactcagccttcagacag -3′ and reverse primer 5′ ggaaagacgaaggcacagag -3′; SDC2 forward primer 5′- tcgagagcagagctgacatc-3′ and reverse primer *5*′*- gcgttctccaaggtcatagc-3*′; SDC3 forward primer 5′- gactcctttcccgatgatga -3′ and reverse primers 5′- gtcagtgggagaggcagaag -3′; SDC4 forward primer 5′- cattaaaccccttccccagt -3′ and reverse primer 5′- agcctgaagaaagcaaacca -3′; IGFBP-3 forward primer 5′-ctgactctgctggtgctgct-3′ and reverse primer 5′-tacggcagggaccatattct-3′; Mknk2 forward primer: *5′- caagaagaggggcaagaaga-3′* and reverse primer: *5′- agtccccgttgagtttgatg-3′*;. PCR reactions were carried in a PTC-200 Peltier Thermal Cycler. Initial denaturation was at 95°C for 4 min and then 35 cycles of denaturation at 95°C for 45 seconds, annealing at 57°C for 30 seconds and extension at 68°C for 60 seconds. The PCR products were separated by electrophoresis on 1% agarose gels and visualized with ethidium bromide staining.

### Immunoblotting

Cells were washed twice with 1x PBS, scraped in 2X- sodium dodecyl sulfate (SDS) sample buffer, boiled, and equal protein amounts were resolved on 10% sodium dodecyl sulfate polyacrylamide (SDS-PAGE) gels. Proteins were transferred to nitrocellulose membranes (Whatman, Germany) and non-specific binding was blocked with 5% nonfat dry milk in TBS-Tween 20 for at least 1 h. Membranes were incubated with primary antibodies overnight at 4°C and horseradish peroxidase-conjugated secondary antibodies for 1 h at room temperature. Signals were detected with enhanced chemiluminescence (Perkin Elmer Life Sciences, Inc., Boston, MA) and autoradiography on X-ray film (Kodak).

### *Ex vivo* human skin assays

Human skin was obtained from corrective plastic surgery. All tissues were obtained according to the guidelines of the University of Pittsburgh and under a protocol approved by the Institutional Review Board of the University of Pittsburgh. As previously described [12], subcutaneous fat tissue was removed uniformly and skin tissue was cut into 1.5 cm×1.5 cm sections. The following were injected intradermally in a total volume of 100 μl: Control adenovirus (Cad), adenovirus expressing human IGFBP-3 (Ad3), or adenovirus expressing human Mknk2a. The preparation of the adenoviral constructs was previously described [8]. Skin tissues were harvested one week post-adenoviral administration, fixed in 10% formalin, and embedded in paraffin.

### Immunohistochemistry

Six µm sections of paraffin-embedded skin and lung tissues were de-paraffinized and antigens retrieved using 10 mM sodium citrate, pH 6.0. Endogenous peroxidase was quenched using 3% Hydrogen peroxide. Sections were blocked with 5% serum and incubated with anti-Syndecan-2, anti-fibronectin antibody, or control antibody (Lab Vision Thermo Scientific, Kalamazoo, MI), followed by secondary antibody. Bound secondary antibody was detected using the AEC Red kit. A light hematoxylin counter stain was used to identify nuclei. Images were taken on a Nikon Eclipse 800 microscope (Nikon Instruments, Inc, Huntley, IL) using identical camera settings.

### Plasmid and Adenovirus constructs

The full-length cDNA encoding Mknk2a was obtained by reverse transcription-polymerase chain reaction (RT-PCR) using total RNA extracted from primary human lung fibroblasts with the following primers: Forward Primer: *5′- cggacagaagATGGTGCAGa-3′* and Reverse Primer:*5′- AGGGTCAggcgtggtctc-3′* (capital letters indicate the coding frame). The cDNA was ligated into the p-GEMTeasy vector (Promega). The reading frame was confirmed by sequence analysis using SP6 and T7 primers. The Mknk2a cDNA was subcloned into the shuttle vector pAdlox and used for the preparation of replication deficient serotype 5 adenovirus expressing Mknk2a in the Vector core facility of the University of Pittsburgh as previously described [8].

### Fibroblast Transfection

MRC-5 fibroblasts were cultured in DMEM supplemented with 10%FBS. Cells were transfected with 1µg DNA (pAdlox-Mknk2a) using Lipofectamine 2000 (Invitrogen Life Technologies, Inc.) following the manufacturer's suggestions.

## Supporting Information

Figure S1 **Silencing IGFBP-3 does not modulate levels of SDC1, SDC3, or SDC4.** Normal fibroblasts were transfected with siRNA targeting IGFBP-3 (siBP3), then stimulated with TGFβ (10 ng/ml) for 48 hours. RT-PCR was used for the detection of SDC1, 3, and 4. β-actin was detected as a loading control.
(TIFF)

Figure S2 **IGFBP-3 does not induce SDC1, 3, or 4 expression.** Primary fibroblasts were infected with Ad-IGFBP3 (Ad3) or control Ad (Cad) at an MOI of 50 for 24 h, 48 h, 72 h and 96 h respectively. SDC1, 3, and 4 gene expression was examined by RT-PCR. β-actin was detected as a loading control.
(TIFF)

Figure S3 **Silencing SDC2 does not alter TGFβ induction of fibrotic genes.** Primary fibroblasts were transfected with control siRNA or SDC2-specific siRNA. After 16 hours, cells were serum starved and treated with vehicle or TGFβ for 48 hrs. Levels of SDC2 mRNA were detected by RT-PCR (**A**) and protein levels of SDC2, Collagen, Fibronectin, CTGF, and αSMA were detected by immunoblotting (**B**). The experiments were repeated in primary human fibroblasts from two different control donors, NL1 and NL2, and MRC-5. GAPDH was detected as a loading control for both mRNA and protein.
(TIFF)

## References

[1] Blobe GC, Schiemann WP, Lodish HF (2000) Role of transforming growth factor beta in human disease. N Engl J Med 342: 1350–8. 10793168 10.1056/NEJM200005043421807

[2] Leask A, Abraham DJ (2004) TGF-beta signaling and the fibrotic response. FASEB J 18: 816–27. 15117886 10.1096/fj.03-1273rev

[3] Martin JL, Baxter RC (1991) Transforming growth factor-beta stimulates production of insulin-like growth factor-binding protein-3 by human skin fibroblasts. Endocrinology 128: 1425–1433. 1705505 10.1210/endo-128-3-1425

[4] Oh Y, Muller HL, Ng L, Rosenfeld RG (1995) Transforming growth factor-beta-induced cell growth inhibition in human breast cancer cells is mediated through insulin-like growth factor-binding protein-3 action. J Biol Chem 270: 13589–13592. 7539790 10.1074/jbc.270.23.13589

[5] Gucev ZS, Oh Y, Kelley KM, Rosenfeld RG (1996) Insulin-like growth factor binding protein 3 mediates retinoic acid- and transforming growth factor beta2-induced growth inhibition in human breast cancer cells. Cancer Res 56: 1545–1550. 8603400

[6] Hwa V, Oh Y, Rosenfeld RG (1997) Insulin-like growth factor binding protein-3 and -5 are regulated by transforming growth factor-beta and retinoic acid in the human prostate adenocarcinoma cell line PC-3. Endocrine 6: 235–242. 9368678 10.1007/BF02820498

[7] Rajah R, Valentinis B, Cohen P (1997) Insulin-like growth factor IGF-binding protein-3 induces apoptosis and mediates the effects of transforming growth factor-beta1 on programmed cell death through a p53- and IGF-independent mechanism. J Biol Chem 272: 12181–12188. 9115291 10.1074/jbc.272.18.12181

[8] Pilewski JM, Liu L, Henry AC, Knauer AV, Feghali-Bostwick CA (2005) Insulin-like growth factor binding proteins 3 and 5 are overexpressed in idiopathic pulmonary fibrosis and contribute to extracellular matrix deposition. Am J Pathol 166: 399–407. 15681824 10.1016/S0002-9440(10)62263-8 PMC1602317

[9] Kuemmerle JF, Murthy KS, Bowers JG (2004) IGFBP-3 activates TGF-beta receptors and directly inhibits growth in human intestinal smooth muscle cells. Am J Physiol Gastrointest Liver Physiol 287: G795–G802. 15178549 10.1152/ajpgi.00009.2004

[10] Lee KW, Liu B, Ma L, Li H, Bang P, et al (2004) Cellular internalization of insulin-like growth factor binding protein-3: distinct endocytic pathways facilitate re-uptake and nuclear localization. J Biol Chem 279: 469–476. 14576164 10.1074/jbc.M307316200

[11] Aston C, Jagirdar J, Lee TC, Hur T, Hintz RL, et al (1995) Enhanced insulin-like growth factor molecules in idiopathic pulmonary fibrosis. Am J Respir Crit Care Med 151: 1597–1603. 7537587 10.1164/ajrccm.151.5.7537587

[12] Yasuoka H, Larregina AT, Yamaguchi Y, Feghali-Bostwick CA (2008) Human skin culture as an *ex vivo* model for assessing the fibrotic effects of insulin-like growth factor binding proteins. Open Rheumatol J 2: 17–22. 19088866 10.2174/1874312900802010017 PMC2577950

[13] Yasuoka H, Jukic DM, Zhou Z, Choi AM, Feghali-Bostwick CA (2006) Insulin-like growth factor binding protein 5 induces skin fibrosis: A novel murine model for dermal fibrosis. Arthritis Rheum 54: 3001–3010. 16947625 10.1002/art.22084

[14] Venkatesan N, Roughley PJ, Ludwig MS (2002) Proteoglycan expression in bleomycin lung fibroblasts: role of transforming growth factor-beta1 and interferon-gamma. Am J Physiol Lung Cell Mol Physiol 283: L806–L814. 12225958 10.1152/ajplung.00061.2002

[15] Sebestyén A, Gallai M, Knittel T, Ambrust T, Ramadori G, et al (2000) Cytokine regulation of syndecan expression in cells of liver origin. Cytokine 12: 1557–1560. 11023673 10.1006/cyto.2000.0754

[16] Worapamorn W, Haase HR, Li H, Bartold PM (2001) Growth factors and cytokines modulate gene expression of cell-surface proteoglycans I human periodontal ligament cells. J Cell Physiol 186: 448–456. 11169984 10.1002/1097-4652(2001)9999:9999<000::AID-JCP1047>3.0.CO;2-V

[17] Worapamorn W, Tam SP, Li H, Haase HR, Bartold PM (2002) Cytokine regulation of syndecan-1 and -2 gene expression in human periodontal fibroblasts and osteoblasts. J Periodontal Res 37: 273–278. 12200971 10.1034/j.1600-0765.2002.01610.x

[18] Waskiewicz AJ, Flynn A, Proud CG, Cooper JA (1997) Mitogen-activated protein kinases activate the serine/threonine kinases Mnk1 and Mknk2. EMBO J 168: 1909–20. 10.1093/emboj/16.8.1909 PMC1169794 9155017

[19] Bernfield M, Kokenyesi M, Kato M, Hinkes MT, Spring J, et al (1992) Biology of the syndecans: a family of transmembrane heparin sulfate proteoglycans. Annu Rev Cell Biol 8: 365–393. 1335744 10.1146/annurev.cb.08.110192.002053

[20] Bernfield M, Gotte M, Park PW, Reizes O, Fitzgerald ML, et al (1999) Functions of cell surface heparan sulphate proteoglycans. Annu Rev Biochem 68: 729–777. 10872465 10.1146/annurev.biochem.68.1.729

[21] Whiteford JR, Behrends V, Kirby H, Kusche-Gulberg M, Maramtsu T, et al (2007) Syndecans promote integrin mediated adhesion of mesenchymal cells in two distinct pathways. Exp Cell Res 313: 3902–3013. 17870067 10.1016/j.yexcr.2007.08.002

[22] Klass CM, Couchman JR, Woods A (2000) Control of extracellular matrix assembly by syndecan-2 proteoglycan. J Cell Sci 113: 493–506. 10639336 10.1242/jcs.113.3.493

[23] Galante LL, Schwarzbauer JE (2007) Requirements for sulfate transport and the diastrophic dysplasia sulfate transporter in fibronectin matrix assembly. JCB 179: 999–1009. 18056413 10.1083/jcb.200707150 PMC2099202

[24] Chen L, Klass C, Woods A (2004) Syndecan-2 regulates transforming growth factor-beta signaling. J Biol Chem 279: 15715–15718. 14976204 10.1074/jbc.C300430200

[25] Cevikbas F, Schaefer L, Uhlig P, Robenek H, Theilmeier G, et al (2008) Unilateral nephrectomy leads to up-regulation of syndecan-2- and TGF-beta-mediated glomerulosclerosis in syndecan-4 deficient male mice. Matrix Biol 27: 42–52. 17681770 10.1016/j.matbio.2007.07.003

[26] Choi S, Kim Y, Park H, Han IO, Chung E, et al (2009) Syndecan-2 overexpression regulates adhesion and migration through cooperation with integrin alpha2. Biochem Biophys Res Commun 384: 231–235. 19394307 10.1016/j.bbrc.2009.04.093

[27] Wang Z, Collighan RJ, Gross SR, Danen EH, Orend G, et al (2010) RGD-independent cell adhesion via a tissue transglutaminase-fibronectin matrix promotes fibronectin fibril deposition and requires syndecan-4/2 and alpha5-beta1 integrin co-signaling. J Biol Chem 285: 40212–40229. 20929862 10.1074/jbc.M110.123703 PMC3001003

[28] Martin JL, Jambazov S (2006) Insulin-like growth factor binding protein-3 in extracellular matrix stimulates adhesion of breast epithelial cells and activation of p44/42 mitogen-activated protein kinase. Endocrinol 147: 4400–4409. 10.1210/en.2006-0094 16763062

[29] Scheper GC, Morrice NA, Kleijn M, Proud CG (2001) The mitogen-activated protein kinase signal-integrating kinase Mknk2 is a eukaryotic initiation factor 4E kinase with high levels of basal activity in mammalian cells. Mol Cell Biol 21: 743–754. 11154262 10.1128/MCB.21.3.743-754.2001 PMC86666

[30] Knauf U, Tschopp C, Gram H (2001) Negative regulation of protein translation by mitogen-activated protein kinase-interacting kinases 1 and 2. Mol Cell Biol 21: 5500–5511. 11463832 10.1128/MCB.21.16.5500-5511.2001 PMC87272

[31] Ueda T, Watanabe-Fukunaga R, Fukuyama H, Nagata S, Fukunaga R (2004) Mknk2 and Mnk1 are essential for constitutive and inducible phosphorylation of eukaryotic initiation factor 4E but not for cell growth or development. Mol Cell Biol 24: 6539–6549. 15254222 10.1128/MCB.24.15.6539-6549.2004 PMC444855

[32] Ryu HY, Lee J, Yang S, Park H, Choi S, et al (2009) Syndecan-2 functions as a docking receptor for pro-matrix metalloproteinase-7 in human colon cancer cells. J Biol Chem 18: 35692–35701. 10.1074/jbc.M109.054254 PMC2791000 19858218

[33] Huang CC, Chuang JH, Chou MH, Wu CL, Chen CM, et al (2005) Matrilysin MMP-7 is a major matrix metalloproteinase upregulated in biliary atresia-associated liver fibrosis. Mod Pathol 18: 941–950. 15696117 10.1038/modpathol.3800374

[34] Fujishima S, Shiomi T, Yamashita S, Yogo Y, Nakano Y, Inoue T, et al (2010) production and activation of matrix metalloproteinase 7 matrilysin 1 in the lungs of patients with idiopathic pulmonary fibrosis. Arch Pathol Lab Med 134: 1136–1142. 20670133 10.5858/2009-0144-OA.1

[35] Munesue S, Yoshitomi Y, Kusano Y, Koyama Y, Nishiyama A, et al (2007) A novel function of syndecan-2, suppression of matrix metalloproteinase-2 activation, which causes suppression of metastasis. J Biol Chem 282: 28164–28174. 17623663 10.1074/jbc.M609812200

[36] Essner JJ, Chen E, Ekker SC (2006) Syndecan-2. Int J Biochem Cell Biol 38: 152–156. 16198615 10.1016/j.biocel.2005.08.012

[37] Park H, Kim Y, Lim Y, Han I, Oh ES (2002) Syndecan-2 mediates adhesion and proliferation of colon carcinoma cells. J Biol Chem 277: 29730–29736. 12055189 10.1074/jbc.M202435200

[38] Villena J, Berndt C, Granés F, Reina M, Vilaró S (2003) Syndecan-2 expression enhances adhesion and proliferation of stably transfected Swiss 3T3 cells. Cell Biol Int 27: 1005–1010. 14642532 10.1016/j.cellbi.2003.09.004

[39] Bayer-Garner IB, Sanderson RD, Dhodapkar MV, Owens RB, Wilson CS (2001) Syndecan-1 CD138 immunoreactivity in bone marrow biopsies of multiple myeloma: shed syndecan-1 accumulates in fibrotic regions. Mod Pathol 14: 1052–1058. 11598177 10.1038/modpathol.3880435

[40] Chen Y, Shi-Wen X, van Beek J, Kennedy L, McLeod M (2005) Matrix contraction by dermal fibroblasts requires transforming growth factor-beta/activin-linked kinase 5, heparan sulfate-containing proteoglycans, and MEK/ERK: insights into pathological scarring in chronic fibrotic disease. Am J Pathol 167: 1699–1711. 16314481 10.1016/s0002-9440(10)61252-7 PMC1613194

[41] Kliment CR, Englert JM, Gochuico BR, Yu G, Kaminski N (2009) Oxidative stress alters syndecan-1 distribution in lungs with pulmonary fibrosis. J Biol Chem 284: 3537–3545. 19073610 10.1074/jbc.M807001200 PMC2635035

[42] Frangogiannis NG (2010) Syndecan-1: a critical mediator in cardiac fibrosis. Hypertension 55: 233–235. 20048190 10.1161/HYPERTENSIONAHA.109.147256 PMC2819313

[43] Jiang D, Liang J, Campanella GS, Guo R, Yu S, et al (2010) Inhibition of pulmonary fibrosis in mice by CXCL10 requires glycosaminoglycan binding and syndecan-4. J Clin Invest 120: 2049–2057. 20484822 10.1172/JCI38644 PMC2877927

[44] Sadikot RT, Christman JW, Blackwell TS (2004) Molecular targets for modulating lung inflammation and injury. 5: 581–588. 10.2174/1389450043345281 15270205

