# Supplementary figures and images for: Syndecan-2 Is a Novel Target of Insulin-Like Growth Factor Binding Protein-3 and Is Over-Expressed in Fibrosis
Source: PLoS One. 2012 Aug 10;7(8):e43049. doi: 10.1371/journal.pone.0043049 (PMC3416749; doi:10.1371/journal.pone.0043049)

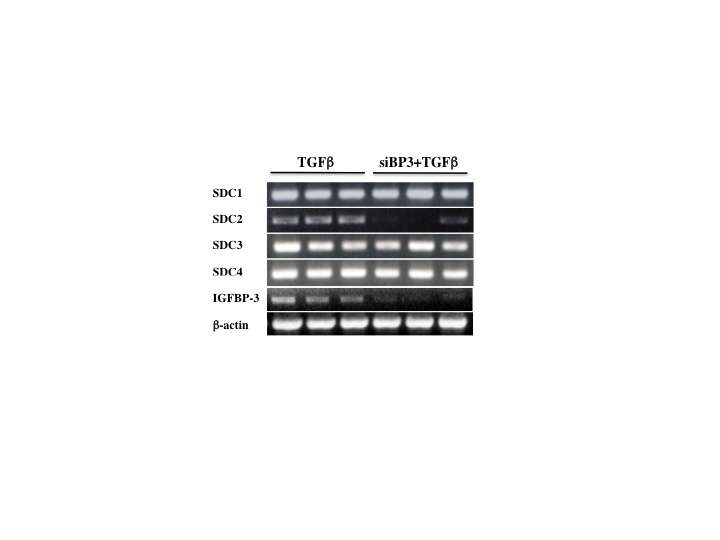

Supplement: Figure S1 — Silencing IGFBP-3 does not modulate levels of SDC1, SDC3, or SDC4. Normal fibroblasts were transfected with siRNA targeting IGFBP-3 (siBP3), then stimulated with TGFβ (10 ng/ml) for 48 hours. RT-PCR was used for the detection of SDC1, 3, and 4. β-actin was detected as a loading control. (TIFF) [file pone.0043049.s001.tiff]

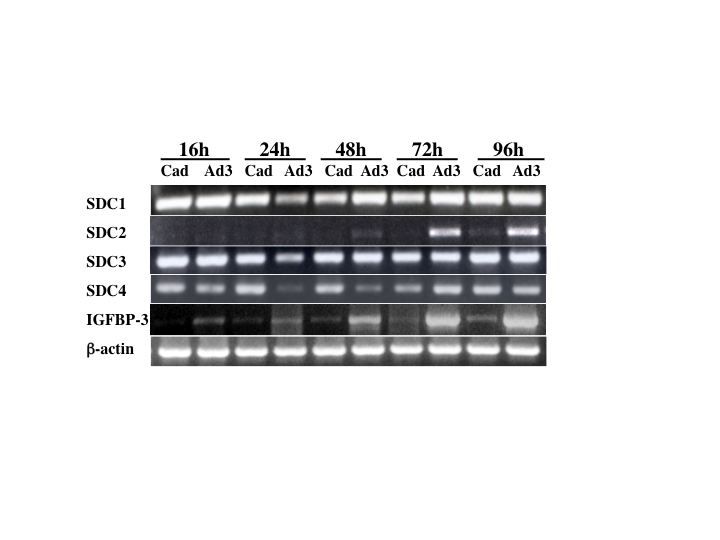

Supplement: Figure S2 — IGFBP-3 does not induce SDC1, 3, or 4 expression. Primary fibroblasts were infected with Ad-IGFBP3 (Ad3) or control Ad (Cad) at an MOI of 50 for 24 h, 48 h, 72 h and 96 h respectively. SDC1, 3, and 4 gene expression was examined by RT-PCR. β-actin was detected as a loading control. (TIFF) [file pone.0043049.s002.tiff]

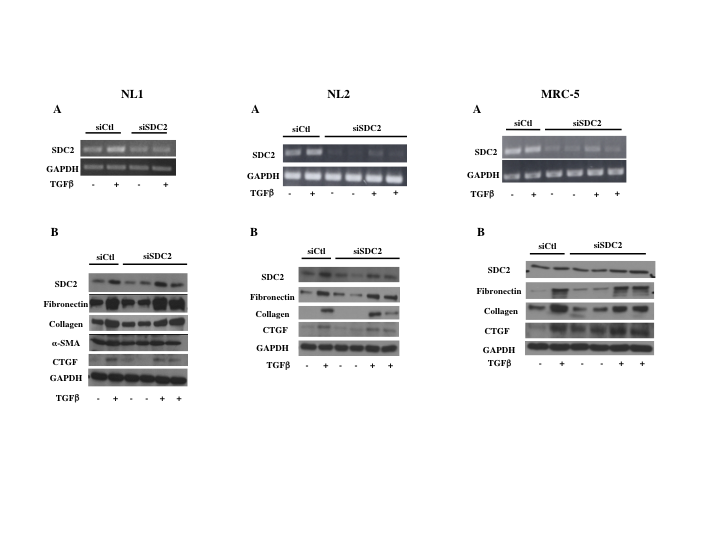

Supplement: Figure S3 — Silencing SDC2 does not alter TGFβ induction of fibrotic genes. Primary fibroblasts were transfected with control siRNA or SDC2-specific siRNA. After 16 hours, cells were serum starved and treated with vehicle or TGFβ for 48 hrs. Levels of SDC2 mRNA were detected by RT-PCR (A) and protein levels of SDC2, Collagen, Fibronectin, CTGF, and αSMA were detected by immunoblotting (B). The experiments were repeated in primary human fibroblasts from two different control donors, NL1 and NL2, and MRC-5. GAPDH was detected as a loading control for both mRNA and protein. (TIFF) [file pone.0043049.s003.tiff]
